# A case of leukemia cutis showing annular erythema during the course of Philadelphia chromosome‐positive acute B‐lymphoblastic leukemia

**DOI:** 10.1002/ccr3.8474

**Published:** 2024-02-09

**Authors:** Hizuru Tomita, Yoshimasa Nobeyama, You Sakayori, Rika Matsumoto, Satomi Chujo, Hikaru Suzuki, Akihiko Asahina

**Affiliations:** ^1^ Department of Dermatology The Jikei University School of Medicine Tokyo Japan; ^2^ Department of Oncology/Hematology The Jikei University School of Medicine Tokyo Japan

**Keywords:** acute B‐lymphoblastic leukemia, annular erythema, blinatumomab, leukemia cutis

## Abstract

We report a case of leukemia cutis showing annular erythema during the course of Philadelphia chromosome‐positive acute B‐lymphoblastic leukemia. The annular appearance may be developed by immunomodulatory effects of blinatumomab.

## 1 INTRODUCTION

Leukemia cutis (LC) is a cutaneous disease caused by the infiltration of neoplastic leukocytes into the skin. The cutaneous manifestations of LC commonly present as patches of homogeneous erythema, papules, and nodules.^1^ Only one case showing annular erythema as LC due to T‐cell acute lymphocytic leukemia (T‐ALL) has been reported to date.^2^ That report did not describe the developmental mechanisms of the cutaneous manifestations. Furthermore, there has been no case showing annular erythema as LC due to B‐cell ALL (B‐ALL).

Blinatumomab is an antibody drug that mediates the formation of a synapse between T‐cells presenting the CD3 antigen and tumor cells presenting the CD19 antigen, resulting in the redirected lysis of CD19‐positive B‐ALL cells.^3^ A phase 2 clinical trial showed that the number of neoplastic leukocytes (tumor cells) decreased below the detection limit within a few days and this effect persisted during the period of blinatumomab treatment.^3^ This trial concomitantly demonstrated that the number of cytotoxic T‐cells rapidly decreased within 1 day after the administration of blinatumomab and recovered above the base number within 1 week, the so‐called redistribution phenomenon.^3^ Although immune‐modulating drugs, such as blinatumomab, potentially affect the cutaneous manifestations of LC through cytotoxic T‐cells, limited information is currently available.

We herein report a case of B‐ALL‐induced LC with the annular appearance of multiple erythema potentially modified by blinatumomab.

## 2 CASE REPORT

A 64‐year‐old man was referred to our Department of Dermatology with a 1‐day history of annular erythema on his trunk. The patient was diagnosed with Philadelphia chromosome‐positive B‐ALL based on the World Health Organization criteria 9 months previously. In this criteria, Philadelphia chromosome‐positive B‐ALL is referred to as B‐lymphoblastic leukemia with t(9;22)(q34.1;q11.2);BCR‐ABL‐1. The diagnosis is based on the bone marrow test to determine blast ratio, the genetic test to investigate chromosomes and the surface marker analysis by flow cytometry. In our case, the bone marrow test showed positive results for CD79α, CD10, and terminal deoxynucleotidyl transferase (TdT) antigens in leukemia cells. The patient was treated with combination chemotherapy consisting of cyclophosphamide, vincristine, doxorubicin, and dexamethasone, resulting in complete remission, followed by consolidation therapy with dasatinib. However, the recurrence of B‐ALL was confirmed 5 months previously based on the detection of bcr/abl mRNA of 4,100,000 copies/μg RNA by a real‐time polymerase chain reaction. Therefore, the patient was referred to our Department of Oncology/Hematology and was treated with blinatumomab (1 course of 42 days; 9 μg/day on Days 1 to 7, 28 μg/day on Days 8 to 28, and washout on days 29 to 42).

**FIGURE 1:**
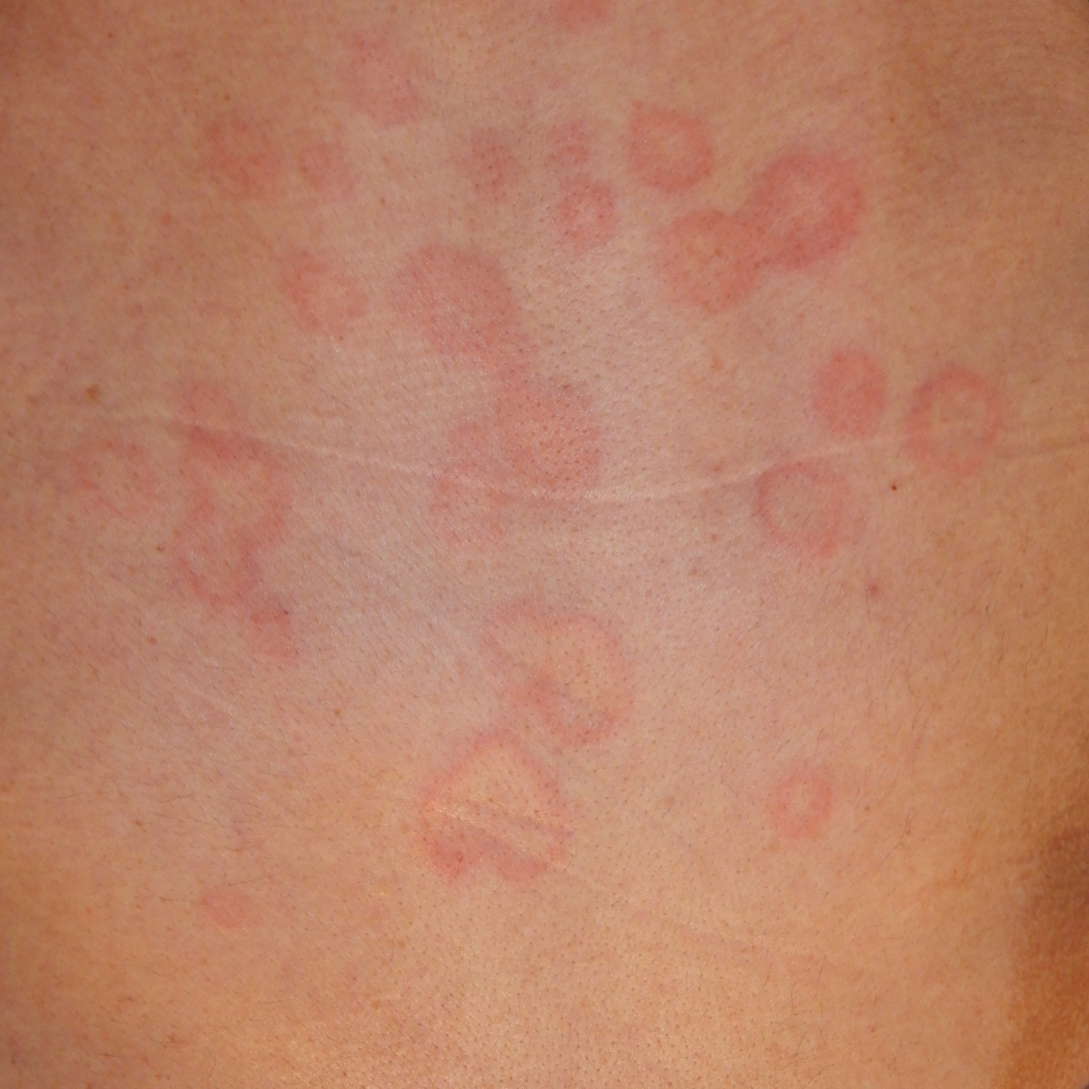
Clinical manifestations in the first visit. Erythema of various sizes with an annular appearance are scattered on the trunk.

**FIGURE 2:**
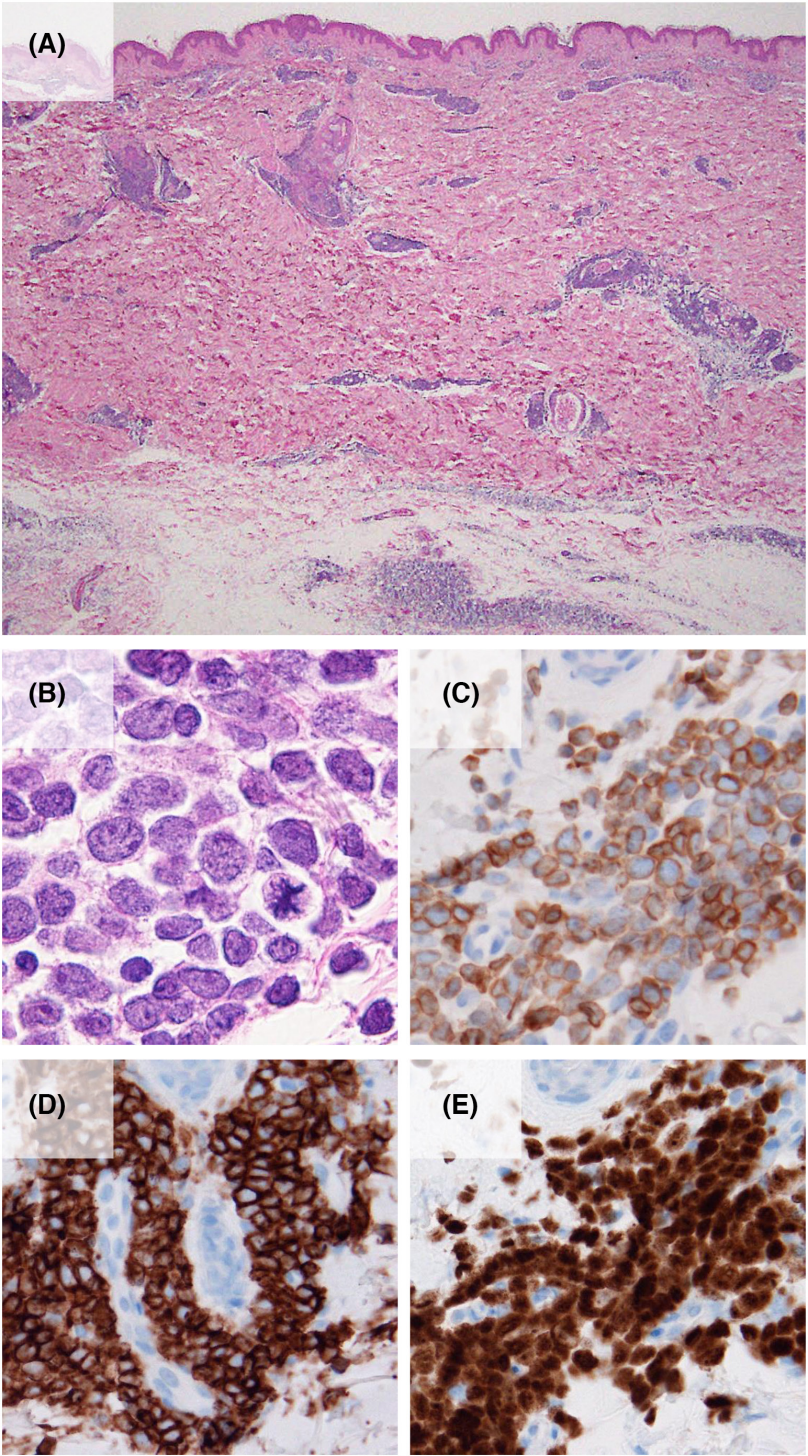
Histopathological findings of annular erythema. (A) A monotonous dense infiltration is observed around vessels and adnexa in the dermis and subcutaneous tissue (hematoxylin–eosin stain, loupe image). (B) Mononuclear cells with dense chromatin and mild atypia in the nucleus are present. A few mitotic cells are observed (hematoxylin–eosin stain, ×1000). (C–E) Immunohistochemical analysis (×400). Infiltrating cells are reactive to the anti‐CD79α antibody (C), CD10 (D), and terminal deoxynucleotidyl transferase (E).

On Day 5 in the 4th course of blinatumomab, the patient developed erythema on his trunk. On Day 6, he visited our Department of Dermatology for the eruption and we noted multiple erythema with an annular appearance and ranging in size from 2 to 5 cm in diameter on his trunk (Figure 1). A histopathological examination of erythema showed that mononuclear cells with dense chromatin and mild atypia infiltrated the nucleus around the vessels and adnexa in the dermis and a few mitotic cells were present (Figure 2A,B). An immunohistochemical analysis revealed that infiltrating mononuclear cells were reactive to anti‐CD79α, CD10, and TdT antibodies (Figure 2C–E, respectively), which was compatible with the bone marrow findings observed 9 months previously. Cutaneous manifestations were diagnosed as LC due to B‐ALL. The manifestations disappeared on Day 7 in the 4th course when it was on the second day after the initiation of topical betamethasone butyrate propionate, leaving only pale pigmentation.

## 3 DISCUSSION

The mechanisms underlying the annular appearance of LC remain unknown; however, an immune reaction against tumor cells may be associated with this phenotype. Previous cases of the skin metastasis of breast cancer showed annular erythema.^4^, ^5^, ^6^ The underlying mechanisms have been explained as follows: (i) tumor cell embolization into the blood and lymph vessels result in local blood congestion and edema, leading to patches of homogeneous erythema centered around emboli; (ii) emboli consisting of tumor cells are attacked by cytotoxic T‐cells and emboli resolve; (iii) blood congestion and edema are sequentially released from the center to the edge of erythema, resulting in the development of the annular appearance of erythema. In the present case, blinatumomab, which may cause an immune reaction against tumor cells, was administered. Immunosuppression may have been induced by the rapid decrease in cytotoxic T‐cells immediately after the initiation of the 4th course, leading to tumor cell embolization and the development of patches of homogeneous erythema as LC. Immediately after this condition, the number of anti‐tumor cytotoxic T‐cells may have increased and these cells may have attacked tumor emboli located in the center of LC, resulting in the annular appearance of erythema temporally.

The present case provides evidence to support the potential involvement of immune‐modulating drugs in the phenotype of annular erythema through cytotoxic T‐cell reactions against tumor cells. Oncologists and dermatologists need to consider characteristic cutaneous adverse events, particularly in patients treated with immune‐modulating drugs.

## AUTHOR CONTRIBUTIONS

**Hizuru Tomita:** Data curation; resources; writing – original draft. **Yoshimasa Nobeyama:** Conceptualization; project administration; writing – review and editing. **You Sakayori:** Resources. **Rika Matsumoto:** Resources. **Satomi Chujo:** Resources. **Hikaru Suzuki:** Resources. **Akihiko Asahina:** Writing – review and editing.

## FUNDING INFORMATION

The authors received no financial support for this study.

## CONFLICT OF INTEREST STATEMENT

The authors have no conflicts of interest to declare.

## ETHICS STATEMENT

The study protocol was approved by The Ethics Committee of The Jikei University School of Medicine and the patient provided written informed consent.

## CONSENT

Written informed consent was obtained from the patient to publish this report in accordance with the journal's patient consent policy.

## Data Availability

Additional data sharing is not applicable to this article due to ethical restrictions.
